# Assessment of end user traits and physicochemical qualities of cassava flour: a case of Zombo district, Uganda

**DOI:** 10.1111/ijfs.14940

**Published:** 2021-01-13

**Authors:** Ann Ritah Nanyonjo, Robert Sezi Kawuki, Florence Kyazze, Williams Esuma, Enoch Wembabazi, Dominique Dufour, Ephraim Nuwamanya, Hale Tufan

**Affiliations:** ^1^ National Crops Resources Research Institute (NaCRRI) 9 km Gayaza‐Zirobwe Road, P.O. Box 7084 Kampala Uganda; ^2^ Makerere University P.O Box 7062 Kampala Uganda; ^3^ CIRAD, UMR Qualisud Montpellier F‐34398 France; ^4^ Qualisud, CIRAD, Institut Agro, Univ Montpellier, Avignon Universit_e, Universit_e de La R_eunion Montpellier France; ^5^ Cornell University Ithaca NY 14850 USA

**Keywords:** Cassava, fermentation, men, physicochemical properties, women

## Abstract

Cassava breeding programmes in Uganda do not currently select materials based on flour making quality, explaining in part the low adoption rates of many released varieties. In this study, we describe end user trait preferences, processing qualities and physicochemical properties of cassava flour. We found that higher proportion of women than men showed preference for most attributes of cassava flour quality evaluated in this study. Preference for colour was 66% and 52% among women and men, respectively, while that for stickiness of *Kwon* was 26% (women) and 15% (men). Ease of peeling and stickiness of *Kwon* were key processing traits. Heap fermented flour had higher pasting temperatures, but lower viscosities than sun‐dried flour, and had lower amylose content compared to fresh root starch. The results demonstrate the importance of gender sensitive participatory evaluation of breeding materials, in tandem with physicochemical evaluation during selection of best possible candidate breeding lines.

## Graphical abstract

This study revealed that higher proportions of women than men showed preference for most attributes of cassava flour quality.

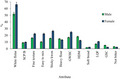

## Introduction

Cassava (*Manihot esculenta* Crantz) is a major root crop grown and consumed in the tropical and sub‐tropical regions of the world (Burns *et al*., 2010). Cassava is mainly cultivated for its starchy roots (Sánchez *et al*., 2009), although its leaves are also eaten as a vegetable (Aduni *et al*., 2005). Cassava roots are bulky and highly perishable, and therefore need to be processed into longer lasting forms such as flour (Bradbury & Denton, 2010; Akama., 2013). Processing also reduces levels of hydrogen cyanide (HCN) (Guédé *et al*., 2013), thus rendering roots of cultivars with high levels of HCN safe for human consumption (Cardoso *et al*., 2005; Lambri *et al*., 2013). In sub‐Saharan Africa, traditional cassava processing methods are highly variable and differentiated geographically. Submerged root fermentation is common practice in Nigeria to produce cassava products such as *gari*, *fufu* and *papuru* (Egwim *et al*., 2016), while heap fermentation is common in Uganda and Mozambique (Alexander *et al*., 1995; Tivana *et al*., 2007).

Cassava breeding efforts have led to identification and deployment of varieties with low HCN content, which are safe for fresh consumption. In Uganda, varietal selection is largely based on defensive and selected agronomic root traits (Kawuki *et al*., 2016). Ideally, roots of such varieties can be processed into flour without undergoing fermentation. However, some communities typically consume cassava products arising from flour processed through fermentation (Cardoso *et al*., 2005; Lambri *et al*., 2013). This narration highlights a major drawback in current cassava breeding operations that are implemented with limited and/or poor understanding of traits that define quality of processed root products.

This challenge is aggravated by commonly used outreach approaches like participatory variety selection that only engage men and women towards the end of variety development process, at which point variability for product quality traits may have been significantly lost or reduced. Consequently, these factors singly and/or in combination have created a situation where genetic gains in cassava for disease resistance, yield and adaptability are high, and on the contrary no and or minimal gains for product traits (Teeken *et al*., 2018).

Essentially, cassava breeding should generate technologies that meet aspirations of men, women, boys and girls; these technologies should also be mindful of their different needs, constraints, roles and responsibilities (Farnworth & Jiggins, 2003). Women perform a bulk of the roles involved in cassava production, processing and marketing (Esuma *et al*., 2019) and thus should be actively involved in technology development, verification and/or dissemination.

For example, Linley *et al*. (2002) found that women continue to grow bitter cassava landraces and not elite varieties owing to the quality superiority of their end products; this has persistent despite the yield and disease susceptibility penalties the landraces experience (Ribeiro *et al*., 2012). It is thus imperative that cassava breeding programmes refocus their breeding strategies to ensure that varieties to be released combine both disease resistance and desirable product quality traits defined by men and women.

Ideally, selection for such traits requires their translation into objectively measureable parameters. Root profiling to quantify starch functional and biophysical properties has been proposed as a suitable strategy to quantify traits of cassava roots (Nuwamanya *et al*., 2010). Indeed, related studies have established that root quality can be assessed by evaluating starch properties and correlating them to amylose content (Nuwamanya *et al*., 2010; Osungbaro *et al*., 2012). Therefore, it is logical to propose that once measurable traits associated with product quality are identified, they can then be used routinely to guide selection. Accordingly, this study was aimed at (i) identifying flour qualities preferred by men and women consumers, (ii) conducting participatory cassava root processing and (iii) evaluating physicochemical properties of elite genotypes and landraces related to cassava flour quality.

## Materials and methods

### Location

This research was conducted in Zombo district, North Western Uganda between 2°30'48"N and 30°54'32"E. Zombo district was chosen because of the unique processing method specific to this area, where cassava roots are initially heap fermented and thereafter processed into flour. In addition, Zombo is a highland area and the cassava breeding programme was targeting to develop varieties suited for such environments.

### Men and women preference of cassava root quality traits

Eight focus group discussions (FGDs) led by a facilitator and guided by preset questions were used to collect qualitative data. Moreover, questionnaires (*n* = 128) were used to collect quantitative data from Sixty‐two men and Sixty‐six women engaged in cassava production. Purposive sampling ensured respondents were from a range of ages, as well as balancing the number of women and men consulted.

### Sources and genotypes of cassava used

The genetic materials used for processing evaluation comprised of 20 elite cassava genotypes sourced from an advanced yield trial and 16 landraces locally grown by men and women in Zombo. The elite genotypes were selected from a highland trial previously conducted in Zombo district in 2016. All agronomic data associated with the highland trial can be accessed at www.cassavabase.org/breeders/trials. An experimental trial was laid in a randomised complete block design with two replicates. Each clone was represented by ten plants per row at a spacing of 1 m × 1 m.

### Processing of cassava roots

Eighteen women from the community were each supplied with cassava roots harvested from four genotypes, to process flour at their homes using the traditional heap fermentation practice with nine women processing roots from one replicate. In each woman’s home, cassava roots were peeled and spread on tarpaulin under the sun. Cassava roots were left under the sun for four to six hours to reduce the moisture content of the roots without complete drying of the roots. Thereafter, whole partially dry roots were heaped in a corner inside the house and covered with polythene sacs. The roots were placed on bare ground in heaps to ferment. Fermentation ended within two to three days, when roots were soft and covered with black moulds. The fermented roots were cleaned with knives to remove black moulds. Clean roots were broken into small pieces by pounding them in a local motor with a local pounding pestle called *Konyu*. Cassava flakes were spread on tarpaulin under the sun until they dried. Dry cassava flakes were pounded with in a local motor with K*onyu* into fine flour. A sieve was used to separate large flakes from fine flour for further pounding. The cassava product *Kwon* was prepared using cassava flour generated following heap fermentation. Briefly, 100 g of cassava flour was mixed with 250 mL of boiling water until a thick paste (referred to as *Kwon*) was formed.

### Evaluation of the processing quality of cassava genotypes

The eighteen women who processed cassava roots evaluated genotypes on attributes related to processing of heap fermented flour and the product *Kwon* (thick paste processed from heap fermented cassava flour). Consequently, a structured questionnaire with questions rated by a scale customised to the Likert scale (Likert, 1932) was used to assess cassava genotypes. Focus was given to attributes, namely (i) ease of peeling assessed by how easily the peel detached from the cortex during peeling; (ii) days to fermentation assessed by the number of days a genotype took to complete the fermentation process; (iii) texture after fermentation assessed by the softness or hardness of the roots after fermentation; (iv) ease of mixing flour with water assessed by how easily the stick moved during mixing cassava flour with boiling water; and (v) quality of cassava *Kwon* evaluated by ability of flour to make a paste that sticks together even when it is left to cool for ten minutes.

### Physical chemical evaluation

Remnant flour from processing evaluation of selected cassava genotypes was used for physicochemical evaluation. Selection of genotypes for physicochemical analyses was based on quality of cassava fermentation (days to fermentation and texture after fermentation) and stickiness of cassava *Kwon*. Quality of fermentation was considered because it is a step in production of cassava flour that influences the characteristics of flour (González & Johnson, 2009). Quality of *Kwon* was considered because the study to determine trait preferences of men and women showed that it was a quality attribute of flour. Therefore, flour from six elite cassava genotypes and six landraces was stored in well labelled air tight containers prior to being subjected to physicochemical evaluation.

Physicochemical analyses were done for heap fermented, sun‐dried and fresh root starch samples. Fresh root starch was prepared following a method described by (Nuwamanya *et al*., 2010). To generate sun‐dried flour, cassava roots were peeled, grated, sun‐dried and pounded with a local motor and pestle. Fresh root starch and sun‐dried samples were packaged in well labelled air tight containers. The samples were evaluated for amylose content, pasting properties, swelling power and solubility. Amylose content of the heap fermented cassava flour and fresh root starch was measured using the method described by (Afoakwa, 2011). Pasting properties of sun‐dried and heap fermented samples were evaluated for using the rapid visco analyser (RVA) profiling procedure described by (Sánchez *et al*., 2010). Swelling power and solubility were determined for fresh root starch using a method described by (Ceballos *et al*., 2007).

### Data analysis

Content analysis described by Elo & Kyngäs (2008) was used to analyse data from FGDs. Briefly, data were examined to generate themes which were used to define cassava product traits. Meanwhile, descriptive statistics were generated from quantitative data using the statistical package for social sciences (SPSS) version 21.0 (2013 release, IBM Corp., Armonk, NY, USA). Summary statistics from evaluation for traits related to processing and the product *Kwon* were obtained using R software version 3.41 (R Core Team, 2020). Similarly, summary statistics from evaluation of physicochemical properties were generated using the same software.

## Results and discussion

### Preference profiles for cassava flour among men and women

This study sought to understand attributes that define the quality of cassava flour processed through heap fermentation, and how preference for such qualities varies among men, women in Zombo district, a highland community in North Western Uganda.

The study showed that most popular cassava landraces processed by both men and women in Zombo are Nyapamitu, Nyarodota and Nyaronega, all of which are bitter (Table 1). Fermentation of cassava roots is not uncommon in communities that cultivate bitter cassava landraces (Linley *et al*., 2015). Due to the inherent toxicity caused by high levels HCN, bitter varieties are often processed to make the resultant product safe and agreeable for consumption (Tivana *et al*., 2007). This means that preference for bitter varieties by men and women in the study may be linked to other attributes such as quality of flour, and not necessarily bitterness. Indeed, the chi‐square test (*x* ^2^ = 9.71, *P *= 0.78, df = 14) indicated that gender and variety processed were independent implying that selection of a variety was largely influenced by its inherent attributes.

**Table 1:** Cassava varieties commonly processed into flour by men and women

Variety	Percentage of men and women who process the variety		Type of variety
	% men (*n *= 62)	%women (*n *= 66)	
Longe	11.3	8.7	Sweet
Nyacharitas	16.3	12.7	Sweet
Nyamatia	11.8	9.2	Bitter
Nyapalei	13.5	10.5	Bitter
Nyapamitu	56.2	43.4	Bitter
Nyapopoga	15.0	12.7	Bitter
Nyaronega	30.4	23.6	Bitter
Nyarudota	48.5	51.5	Bitter

Chi‐square (*x* ^2^ = 9.71, *P* = 0.78, df = 14).

Cassava roots are commonly processed into flour following a stepwise procedure. In Zombo, the procedure of processing roots into flour was by heap fermentation. Apart from reducing levels of HCN, heap fermentation was purported to improve organoleptic attributes of cassava flour.

…fermenting removes bitterness, it gives cassava flour a nice smell and the ability to make sticky food… (Woman, Zeu sub‐county FGD)

According to Putri *et al*. (2011), heap fermentation improves the odour and taste of cassava flour.

**Figure 1:**
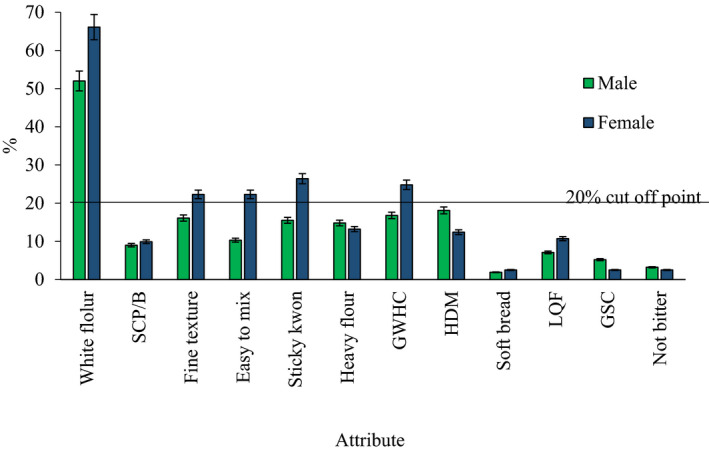
Preferred flour quality attributes of men and women in Zombo district. SCP/B = sweet cassava paste/ flour, GWHC = good water holding capacity, HDM = high dry matter, LQF = large quantities of flour, GSC = good swelling capacity. % is a symbol for percentage.

A range of attributes were mentioned by men and women in describing their perception of high quality cassava flour (Fig. 1). Considering a cut‐off of 20%, some attributes were preferred more by women than by men. Notable of these were white flour (66% women verses 52% men), fine texture (22% women verses 16% men), ease of mixing (22% women verses 10% men), good water holding capacity (24% women verses 16% men) and sticky *Kwon* (26% women verses 15% men), respectively. Despite this, heavy flour and high dry matter content were preferred by more men than women, although they did not meet the cut‐off point. These flour quality attributes preferred by women and men are linked to cassava *Kwon*. This finding highlights both the importance of *Kwon* to the Zombo community and need for participatory product‐based evaluation and selection.

### Processing and physicochemical properties of elite cassava genotypes and landraces

Cassava genotypes used in this study were evaluated for major aspects of the processing procedure. We noted significant genotypic differences (*P* < 0.05) for ease of peeling and stickiness of *Kwon* (Table 2). Elite genotypes had an average of 3.1 for ease of peeling while land races had 3.9 clearly illustrating that landraces scored highly for ease of peeling (Table 3). According to González & Johnson (2009), ease of peeling was one of the quality attributes that consumers considered when selecting cassava roots to purchase.

**Table 2:** Analysis of variance for processing evaluation traits

Variation	df	EoP	DtF	TaF	EoM	SoK
Replicate	1	0.09	2.02	5.40	0.49	0.31
Genotype Error	35 36	2.15^*^ 32.00	0.39 30.00	1.30 30.00	1.88 2.67	1.69^**^ 0.55

df, degrees of freedom; DtF, days to fermentation; EoM, Ease of mixing; EoP, ease of peeling; SoK, stickiness of *Kwon*.

* significant at <0.05.

** siginificant at < 0.01.

**Table 3:** Mean performance of genotypes for sticky cassava *Kwon* and ease of peeling

Genotype	Sticky cassava *Kwon*	Ease of peeling
Elite		
UGH150022	5.0a	3.0bc
UGH150024	5.0a	4.0ab
UGH150031	5.0a	2.5c
UGH150040	5.0a	1.0c
UGH150085	5.0a	4.0ab
UGH150091	5.0a	3.5bc
UGH150094	5.0a	2.0c
UGH150105	5.0a	3.0bc
UGH150461	5.0a	5.0a
UGH150067	4.5ab	3.0bc
UGH150073	4.5ab	5.0a
UGH150014	4.0ab	2.5c
UGH150059	4.0ab	2.0c
UGH150053	3.5bc	2.0c
UGH150155	3.5bc	2.5c
UGH150023	3.0bc	5.0a
UGH150058	3.0bc	3.5bc
UGH150089	2.5c	3.0bc
UGH150121	2.0c	1.5c
Average of elite	4.2	3.1
Local varieties		
Longe	5.0a	4.5ab
Nyamatia	5.0a	5.0a
Nyamukalasa	5.0a	4.5ab
Nyamukele	5.0a	2.0c
Nyapalei	5.0a	3.5bc
Nyarodota	5.0a	3.5bc
Tme 14	5.0a	3.5bc
Nyacharitas	4.5ab	5.0a
Nyalusi	4.5ab	4.5ab
Nyamateo	4.5ab	3.5bc
Akena	4.0ab	4.0ab
Nyamukelele	4.0ab	4.0ab
Nyapopoga	4.0ab	4.0ab
Nyaronega	4.0ab	3.5bc
Nyagota	2.5c	3.0bc
Telengule	2.0c	3.5bc
Nyapamitu	4.5ab	4.0ab
Average of local varieties	4.3	3.9
LSD (5%)	2.05	2.40

Note

Alphabetical letters show grouping of clones after mean separation using Fisher’s protected least significant difference (LSD) test at 5%.

**Figure 2:**
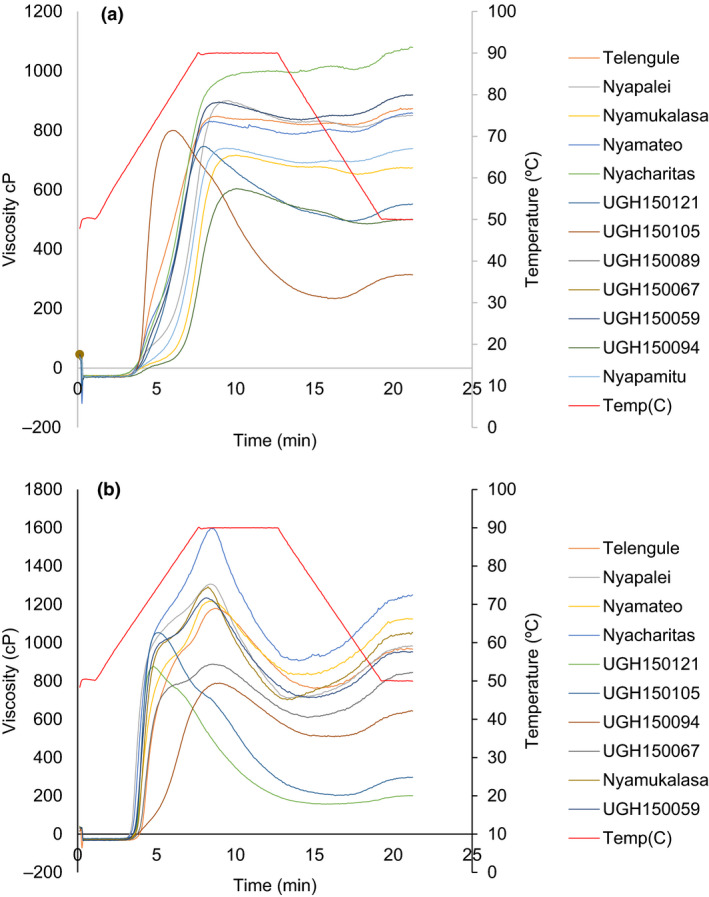
Paste profile of selected cassava genotypes: (a) heap fermented cassava flour; (b) sun‐dried cassava flour. Heap fermented samples maintain paste stability at high temperature.

Furthermore, sticky *Kwon* ranged from 2.0 up to 5.0 (Table 3). It suffices to note that landraces had a slightly higher (4.3) average for sticky *Kwon* than elite genotypes (4.2). The ability of cassava flour to form a ‘thick paste’ referred to as ‘stickiness’ is a critical starch pasting property whose differences are reported to be genotype dependant (Asaoka *et al*., 1992). These differences in starch pasting properties provide breeders an opportunity to make selections (Nuwamanya *et al*., 2010). Starch pasting properties which occur during heating are well illustrated through RVA profiling (Osungbaro *et al*., 2012). Accordingly, we obtained RVA profiles for heap fermented and sun‐dried cassava flour samples (Fig. 2).

Heap fermented flour samples had lower values for peak, final and breakdown viscosities than the sun‐dried flour samples. On the other hand, heap fermented flour samples had higher pasting temperatures and trough viscosity as compared to the sun‐dried flour samples. While sun‐dried flour samples had a characteristic pasting profile with a visible break down, most heap fermented flour samples had unique profiles with no visible break down (Fig. 2). Indeed Gomes *et al*. (2005) found similar observations on fermented cassava starch which was modified by annealing. The authors attributed reduced peak viscosity to decreased amylose leaching.

Annealing is a process that modifies starch by rearranging starch granules without destroying the their structure (Alcázar‐Alay & Meireles, 2015). According to Corke (2007), such modifications result into starch following the type C pasting profile (has no visible pasting peak and breakdown) as were observed from RVA profiles of heap fermented samples in this study. Having no visible breakdown on the pasting profile indicates low extent of breakdown of the paste and thus paste stability at high temperatures (Eriksson, 2013). This attribute has positive implications on the ability of cassava flour to form *Kwon* at high temperature since the paste will remain viscous even at high temperature.

Nyacharitas had the highest peak, trough and end viscosities for sun‐dried flour (Fig. 2b). In addition, Nyacharitast had the highest peak, trough and end viscosities for heap fermented flour (Fig. 2a). It suffices to note that, Nyacharitas had high ability to make sticky cassava *Kwon* (4.5) (Table 3). This finding suggest that high peak viscosities are associated to stickiness, a pre‐condition that has to be examined further with more diverse cassava genotypes. Nyamukalasa (85.35 ºC) and UGH150094 (85.35 ºC) had the highest pasting temperature for heap fermented flour, while UGH150094 (78.95 ºC) had the highest for sun‐dried flour (Table 4). It is likely that high pasting temperature could be due to high amylose content (Ekwu *et al*., 2011).

**Table 4:** Mean genotype performance for pasting temperature

Genotype	PT (ºC)
Fermented UGH150059	82.55 ± 0.60
Sun‐dried UGH150059	65.65 ± 0.00
Fermented UGH150067	84.15 ± 0.03
Sun‐dried UGH150067	67.70 ± 0.00
Fermented UGH150094	85.35 ± 0.00
Sun‐dried UGH150094	78.95 ± 1.13
Fermented UGH150105	67.3 ± 0.21
Sun‐dried UGH150105	65.83 ± 0.24
Fermented UGH150121	78.45 ± 1.94
Sun‐dried UGH150121	66.05 ± 0.07
Fermented Nyacharitas	78.95 ± 0.03
Sun‐dried Nyacharitas	64.76 ± 0.11
Fermented Nyamateo	80.95 ± 0.56
Sun‐dried Nyamateo	66.03 ± 0.11
Fermented Nyamukalasa	85.35 ± 0.00
Sun‐dried Nyamukalasa	65.28 ± 1.09
Fermented Nyapaleyi	83.8 ± 0.28
Sun‐dried Nyapaleyi	64.02 ± 0.04
Fermented Telengule	68.45 ± 0.03
Sun‐dried Telengule	68.05 ± 0.07
LSD fermented	11.55
LSD sun‐dried	2.26

Results for amylose content are presented in Table 5. Genotype UGH150121 had the highest amylose content (30.76) among the fresh starch samples, while UGH150105 had the least amylose (22.1%). With regard to the heap fermented flour samples, Nyamukalasa had the highest amount amylose (22.5%), while Nyacharitas had the lowest (17.0%). Amylose content was higher in fresh root starch samples (with average 25.38) compared to heap fermented flour (with average 20.95). Such differences could be due to amylose degradation by lactic acid bacteria (*Lactobacillus fermentum*) which is a key organism involved in fermentation (Tivana *et al*., 2007). Indeed, amylose content could have implications on pasting properties because it is a non‐branched polymer of starch that is not easily hydrolysed (BeMiller & Whistler, 2009). Consequently, when exposed to heat, cassava flour from Nyamukalasa would require higher temperature to gelatinise and form a thick paste (*Kwon*) than that from Nyacharitas. The relationship between amylose content, pasting properties and stickiness of cassava *Kwon*, if further investigated, could be key in evaluating genotypes for the important attribute – stickiness of cassava *Kwon*.

**Table 5:** Amylose content of fresh root starch and heap fermented flour

Genotype	Amylose content (%)	
	Fresh roots starch	Heap fermented flour
UGH150121	30.76^a^ ± 2.51	21.72^a^ ± 0.82
UGH150059	26.94^a^ ± 0.48	20.53^a^ ± 0.46
UGH150067	22.35^a^ ± 0.67	21.12^a^ ± 4.23
UGH150089	25.49^a^ ± 2.47	19.32^a^ ± 2.06
UGH150105	22.05^a^ ± 5.18	20.63^a^ ± 1.17
UGH150094	28.70^a^ ± 0.71	21.96^a^ ± 0.30
Nyapamitu	23.01^a^ ± 0.89	24.32^a^ ± 0.66
Telengule	23.32^a^ ± 0.08	21.18^a^ ± 1.54
Nyamukalasa	26.01^a^ ± 4.92	22.54^a^ ± 2.17
Nyapalei	23.31^a^ ± 3.36	22.25^a^ ± 1.89
Nyacharitas	23.45^a^ ± 0.61	17.02^a^ ± 5.09
Nyamateo	29.14^a^ ± 2.88	18.91^a^ ± 0.93
Mean	25.38	20.95
LSD at 5%	24.66	12.10
CV (%)	5.07	4.57

CV, coefficient of variation; LSD, least significant difference. Note: Letters show grouping of clones after mean separation using Fisher’s protected least significant difference (LSD) test at 5 %

Alphabetical letters show grouping of clones after mean separation using Fisher’s protected least significant difference (LSD) test at 5%.

**Figure 3:**
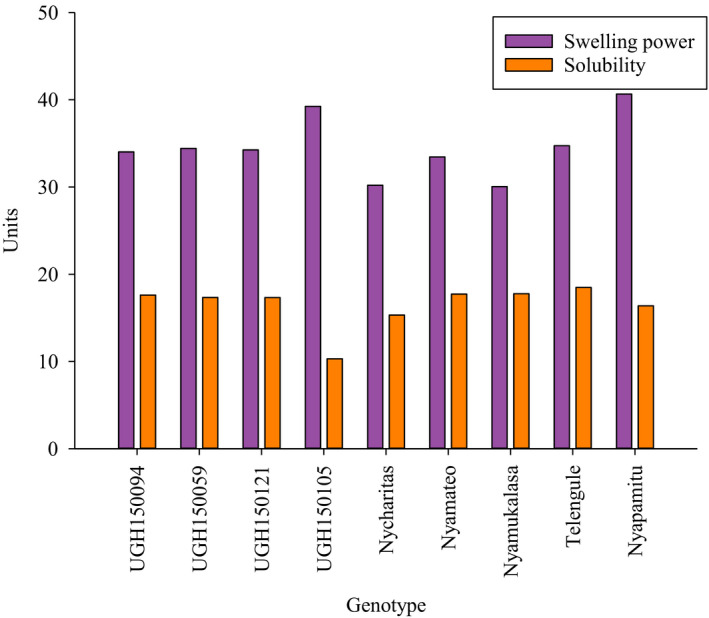
Swelling power and solubility of fresh root cassava starch. Units = swelling power (g of water/ g of starch) and solubility (%).

Meanwhile, swelling power of cassava starch ranged from 30 to 40 g of water/g of starch. Nyapamitu had the highest ability to swell (40. 66 g of water/ g of starch) while Nyacharitas had the lowest swellability of 30.20 g of water/g of starch (Fig. 3). Solubility of fresh root cassava starch ranged from 10 to 18%. Telengule (18.48%) had the highest solubility while UGH150105 (10.30%) had the lowest solubility. According to Nuwamanya *et al*. (2010), swelling ability is inversely related to its amylose content. Indeed, UGH150105 which had the lowest amylose content (22.05%) (Table 5) had high ability to swell (39.25 g of water/ g of starch, Fig. 3). Performance of UGH150105 for swelling power and solubility corroborated the findings by Ceballos *et al*. (2007) on mutant cassava. According to the authors, the mutant genotype had lower solubility but higher swelling ability and they related it to its low amylose content.

## Conclusion

Data sets presented in this study highlight three major findings. First, men and women differ in their cassava flour preferences, strengthening the argument for considering gender related traits during selection, and including women and men to participate in the variety development process. Second, landraces possessed better processing qualities compared to elite genotypes. Thus, land races with superior processing qualities can be used as progenitors and/or checks when under taking root quality assessment. Third, physicochemical properties notably paste properties exhibited strong association with *Kwon* product thickness, a key quality attribute. This information could guide development of protocols for making selections for quality cassava flour. We postulate that this will contribute to development of improved varieties that exhibit critical quality traits preferred by men and women, for increasing adoption of released varieties.

## Conflict of interest

The authors declare that they have no conflict of interests.

## Author contribution

**Ritah Ann Nanyonjo:** Conceptualization (supporting); Data curation (lead); Formal analysis (lead); Investigation (lead); Methodology (equal); Writing‐original draft (lead); Writing‐review & editing (lead). **RoberT Sezi Kawuki:** Conceptualization (equal); Formal analysis (supporting); Funding acquisition (supporting); Investigation (supporting); Methodology (equal); Project administration (lead); Resources (supporting); Supervision (lead); Writing‐review & editing (equal). **Florence Birungi Kyazze:** Data curation (supporting); Formal analysis (supporting); Investigation (supporting); Methodology (equal); Supervision (equal); Writing‐review & editing (equal). **Williams Esuma:** Data curation (supporting); Formal analysis (equal); Methodology (supporting); Writing‐original draft (supporting); Writing‐review & editing (equal). **Enoch Wembabazi:** Data curation (equal); Formal analysis (equal); Investigation (supporting); Writing‐review & editing (equal). **Dominique Dufour:** Conceptualization (equal); Formal analysis (equal); Investigation (supporting); Methodology (equal); Supervision (equal); Writing‐review & editing (equal). **Ephraim Nuwamanya:** Conceptualization (supporting); Data curation (supporting); Formal analysis (equal); Investigation (supporting); Methodology (equal); Supervision (equal); Writing‐review & editing (equal). **Hale Tufan:** Conceptualization (lead); Formal analysis (supporting); Funding acquisition (lead); Investigation (supporting); Methodology (lead); Project administration (supporting); Resources (lead); Writing‐original draft (supporting); Writing‐review & editing (equal).

## Ethical approval

Men and women were asked for their consent to participate and were assured of the anonymity of the information they gave. In addition, introduction of the study and its purpose was made. Indeed, respondents were free to cancel the interview any time if they lost interest to participate.

### Peer review

The peer review history for this article is available at https://publons.com/publon/10.1111/ijfs.14940.

## Data Availability

Data that support the findings of this study are available from the corresponding author upon reasonable request.
